# Editorial: Memory Processes in Medial Temporal Lobe: Experimental, Theoretical and Computational Approaches

**DOI:** 10.3389/fnsys.2017.00019

**Published:** 2017-04-06

**Authors:** Vassilis Cutsuridis, Motoharu Yoshida

**Affiliations:** ^1^School of Computer Science, University of LincolnLincoln, UK; ^2^Leibniz Institute for Neurobiology MagdeburgMagdeburg, Germany; ^3^German Center for Neurodegenerative Diseases (DZNE) MagdeburgMagdeburg, Germany

**Keywords:** memory, memory disorders, computational modeling, medial temporal lobes

The medial temporal lobe (MTL) includes the hippocampus, amygdala and parahippocampal regions, and is crucial for episodic and spatial memory. MTL memory function consists of distinct processes such as encoding, consolidation and retrieval. Encoding is the process by which perceived information is transformed into a memory trace. After encoding, memory traces are stabilized by consolidation. Memory retrieval (recall) refers to the process by which memory traces are reactivated to access information previously encoded and stored in the brain. Although underlying neural mechanisms supporting these distinct functional stages remain largely unknown, recent studies have indicated that distinct oscillatory dynamics, specific neuron types, synaptic plasticity and neuromodulation, play a central role. The theta rhythm is believed to be crucial in the encoding and retrieval of memories. Experimental and computational studies indicate that precise timing of principal cell firing in the hippocampus, relative to the theta rhythm, underlies encoding and retrieval processes. On the other hand, sharp-wave ripples have been implicated in the consolidation through the “replay” of memories in compressed time scales.

The neural circuits and cell types supporting memory processes in the MTL areas have only recently been delineated using experimental approaches such as optogenetics, juxtacellular recordings, and optical imaging. Principal (excitatory) cells are crucial for encoding, storing and retrieving memories at the cellular level, whereas inhibitory interneurons provide the temporal structures for orchestrating the activities of neuronal populations of principal cells by regulating synaptic integration and timing of action potential generation of principal cells as well as the generation and maintenance of network oscillations (rhythms). In addition, neuromodulators such as acetylcholine alter dynamical properties of neurons and synapses, and modulate oscillatory state and rules of synaptic plasticity and their levels might tune MTL to specific memory processes.

The research topic offers a snapshot of the current state of-the-art on how memories are encoded, consolidated, stored and retrieved in MTL structures. Accepted papers to the research topic include studies (experimental or computational) focusing on the structure and function of neural circuits, their cellular components (principal cell and inhibitory interneurons) and their properties, synaptic plasticity rules involved in these memory processes, network oscillations such as theta, gamma and sharp-wave ripples, and the role of neuromodulators in health and in disease (Alzheimer's disease and schizophrenia).

## Cell activity properties in MTL structures

Starting the research topic Niediek and Bain provided a commentary of the Miller et al. (2013) experimental study of place cell activity in human hippocampal formation by means of its spatial context. Although according to Niediek and Bain the Miller et al. (2013) study was an important one in memory research, they discussed several of its shortcomings. They concluded that further human research is needed to clarify the distribution, size and shape of human place-cell fields.

Xie et al. continued by presenting a 512 channel recording technique which allowed the simultaneous recording of both single units and local field potentials from 13 distinct neural circuits of the mouse brain including subregions of the anterior cingulate cortices, retrosplenial cortices, somatosensory cortices, secondary auditory cortex, hippocampal CA1, dentate gyrus, subiculum, lateral entorhinal cortex, perirhinal cortex and prelimbic cortex. The authors demonstrated that complex stimuli—such as earthquake and fear-inducing foot-shock—trigger firing changes in all of the recorded 13 brain regions, supporting the notion that neural code is highly distributed.

## Theta

Theta oscillations play a crucial role in learning and memory. Hoffmann et al. has reviewed so called theta-contingent training where training of the animal is initiated when sufficiently strong theta oscillations have been detected by a brain-computer interface. Theta-contingent training increases the learning speed two- to four times, indicating that the MTL is optimized for learning during theta oscillations. The authors further describe underlying cellular mechanisms for this facilitation of learning in terms of local field potentials, neural firing and possible involvement of the cholinergic system. Long et al. reviewed theta variation and spatiotemporal scaling along the septotemporal axis of the hippocampus. The review highlighted a series of studies examining theta local field potential signals across the septotemporal or longitudinal axis of the HPC. While the theta signal is coherent throughout the entirety of the HPC, the amplitude, but not the frequency, of theta varies significantly across its three-dimensional expanse. The authors offered a working model where they speculated that syncrhonization of theta across the septotemporal axis served to bind ensembles representing varying resolutions of spatiotemporal information at interdependent septotemporal areas of the HPC. Such synchrony and cooperative interactions along the septotemporal axis likely support memory formation and subsequent consolidation and retrieval. Ferguson et al. created network models of the hippocampus containing different classes of inhibitory interneurons in order to explore at the cellular and network levels how these interneuron interactions affect the power of local theta oscillations. They found that OLM cells inactivation result in no change or even an increase in theta power. They predicted that the dis-inhibitory effect of OLM cells to bistratified cells to pyramidal cell interactions played a critical role in the resulting power of network theta oscillations.

Theta oscillations are believed to coordinate encoding and retrieval stages of the MTL based on neuronal firing at different phase of the theta (Hasselmo et al., 2002). In this research topic, Newman and Hasselmo have explored whether grid cells in the medial entorhinal cortex have different characteristics depending on their preferred firing phase. They found that grid cells fired preferentially at the peak or trough of the theta indicating its role in encoding and retrieval, respectively. They further demonstrate that trough-locked grid cells contain more spatial information, stronger head direction tuning, and show stronger phase precession, compared to peak-locked grid cells. These results indicate that peak and trough locked grid cells are two distinct populations and they may serve different functions.

## Associative memories in MTL in health and in disease

Raudies and Hasselmo presented a spiking feedforward network model able to learn the link between item and context while developing independence for place when predicting reward. The model fitted empirical data where item and context selectivity significantly increased while the place selectivity remained constant (Komorowski et al., 2009) and suggested a new learning method based on replaying rewarded sequences in forward temporal order and non-rewarded sequences in backward temporal order.

Carrere and Alexandre present a computational model of pavlovian conditioning in amygdala. The model is grounded on biological data, replicates a number of biological experiments and offers predictions about the role of amygdalar regions and describes pavlovian conditioning as a distributed systemic learning, binding memory processes in the MTL.

Kassab and Alexandre present a computational network model of interconnected auto- and hetero-associative memories that addresses the rapid, one-trial, binding of extero-interoceptive features within the hippocampus. In the model, neural assemblies representing exteroceptive sensory inputs and their emotional valences are considered as two sets of independent features, each stored apart in autoassociative memories that are linked in a heteroassociative way. This implies a distinction between two specific forms of interference, namely “pattern overload” and “valence overload” that can occur respectively at the level of the autoassociative and heteroassociative memories.

Woodcock et al. provided evidence of dysfunctional frontal-medial temporal lobe network signatures in schizophrenia consistent with the disconnection syndrome hypothesis by investigating network profiles of dorsal prefrontal and dorsal anterior cingulate cortices during memory encoding and retrieval in schizophrenia patients and healthy controls. Patients showed a pattern of exaggerated modulation in both frontal regions during memory encoding and retrieval. Furthermore, relatively diminished modulation during encoding was associated with increased modulation during retrieval in regions of the hippocampus.

## Consolidation

Once memory is encoded, consolidation occurs to maintain and strengthen the memory. It is believed that memory is redistributed from the MTL to the neocortex during systems consolidation which occurs during sleep and quiet waking (Dudai et al., 2015). Cordi et al. have investigated whether a presentation of sensory cue which was present during memory encoding would facilitate memory consolidation if the same cue was introduced during slow-wave sleep (SWS) and REM sleep after the encoding stage. As previously demonstrated by the same group (Rasch et al., 2007), presentation of the cue during SWS facilitated memory consolidation. However, there was no effect of cue presentation during REM sleep. These results support the idea that cue induced reactivation (replay) of hippocampal cells support systems consolidation specifically during SWS (Girardeau et al., 2009; Bendor and Wilson, 2012), but not during REM sleep.

## Working memory

It has recently been evident that MTL areas support working memory and temporal association tasks, which require short-term retention of information (Yoshida et al., 2012). Griffin has reviewed the role of thalamic nucleus reuniens in synchronizing oscillatory activity between the hippocampus and medial prefrontal cortex. One neural mechanism supporting working memory is persistent activity (Major and Tank, 2004). MTL neurons including CA1 and CA3 pyramidal cells can support persistent activity under cholinergic receptor activation (Jochems and Yoshida, 2013; Knauer et al., 2013). Using high-resolution fMRI, Nauer et al. have investigated whether persistent activity in the MTL underlies both working memory and encoding of long-term memory. They report that persistent activity in the MTL was lineally related to increases in subsequent memory strength, suggesting that persistent firing was supporting not only working memory but also episodic encoding.

## Aging/AD

The MTL areas are crucially involved in dementia and aging. Klein et al. have demonstrated that neural mechanism supporting gamma oscillations might be compromised in Alzheimer's disease. Using an *in vitro* preparation from a mouse model of Alzheimer [the transgenic (tg) amyloid precursor protein (APP)-presenilin 1 (PS1)], they reported that gamma oscillations are slower and smaller in the lateral but not in the medial entorhinal cortex. This result suggests that signal entering the hippocampus through the medial and lateral entorhinal cortex might not be integrated properly in Alzheimer disease. Aging seems to alter excitability of neurons in a subregion-dependent manner in the hippocampus. Oh et al. have provided a review on reduced and increased excitability in CA1 and CA3 areas, respectively, focusing on the cellular mechanisms underlying this change in aged animals. They further discuss that calcium regulation might be the key for future therapeutics.

We hope this research topic will inspire readers to actively research memory using both experimental and computational techniques. As it was clearly shown in this research topic, a multidisciplinary approach is needed to decipher the mechanisms of memory encoding, retrieval and consolidation, We would like to thank all authors who have provided us with exciting and forefront contributions to this research topic.

## Author contributions

All authors listed, have made substantial, direct and intellectual contribution to the work, and approved it for publication.

### Conflict of interest statement

The authors declare that the research was conducted in the absence of any commercial or financial relationships that could be construed as a potential conflict of interest.
